# Marine Data Prediction: An Evaluation of Machine Learning, Deep Learning, and Statistical Predictive Models

**DOI:** 10.1155/2021/8551167

**Published:** 2021-11-27

**Authors:** Ahmed Ali, Ahmed Fathalla, Ahmad Salah, Mahmoud Bekhit, Esraa Eldesouky

**Affiliations:** ^1^Department of Computer Science, College of Computer Engineering and Sciences, Prince Sattam Bin Abdulaziz University, Al-Kharj 11942, Saudi Arabia; ^2^Higher Future Institute for Specialized Technological Studies, Cairo 3044, Egypt; ^3^Department of Mathematics, Faculty of Science, Suez Canal University, Ismailia 41522, Egypt; ^4^College of Information Science and Engineering, Hunan University, Changsha 410082, China; ^5^Faculty of Computers and Informatics, Zagazig University, Sharkeya 44523, Egypt; ^6^Computer Science Department, Faculty of Computers and Informatics, Suez Canal University, Ismailia 41522, Egypt

## Abstract

Nowadays, ocean observation technology continues to progress, resulting in a huge increase in marine data volume and dimensionality. This volume of data provides a golden opportunity to train predictive models, as the more the data is, the better the predictive model is. Predicting marine data such as sea surface temperature (SST) and Significant Wave Height (SWH) is a vital task in a variety of disciplines, including marine activities, deep-sea, and marine biodiversity monitoring. The literature has efforts to forecast such marine data; these efforts can be classified into three classes: machine learning, deep learning, and statistical predictive models. To the best of the authors' knowledge, no study compared the performance of these three approaches on a real dataset. This paper focuses on the prediction of two critical marine features: the SST and SWH. In this work, we proposed implementing statistical, deep learning, and machine learning models for predicting the SST and SWH on a real dataset obtained from the Korea Hydrographic and Oceanographic Agency. Then, we proposed comparing these three predictive approaches on four different evaluation metrics. Experimental results have revealed that the deep learning model slightly outperformed the machine learning models for overall performance, and both of these approaches greatly outperformed the statistical predictive model.

## 1. Introduction

Forecasting maritime parameters such as wave conditions, tide length, wind direction, rainfall, etc., is of great importance. For example, marine data prediction can help optimize shipping routes by detecting rough seas, coastal and offshore engineering, environmental protection, and planning sea-related activities. These require real-time and short-term prediction of the ocean marine data for the following hours and next few days.

SST means the ocean's surface temperature. The forecasting of SST is considered an essential task in various real-life situations (e.g., ocean weather and climate prediction, fishing, and ocean environment protection). In this task, the predictive model produces its future SST value in advance (e.g., minutes or hours); thus, this predicted value can improve the decisions related to several activities such as fishing and maritime navigation. Similarly, SWH refers to the significant wave height of oceans. Knowing the SWH in advance is important for several maritime activities such as surfing and maritime navigation. Predictive models can predict the expected SWH based on the historical SWH in a particular geographical maritime area.

The dimensions of ocean marine data are rapidly increasing. Furthermore, the vast majority of the ocean's big data is unstructured or semistructured, with complex or irrelevant relationships between the data, revealing many shortcomings in traditional data analysis approaches. These shortcomings have been addressed by developing machine learning (ML) models, which have proven to be robust, fast, and highly accurate [1]. For instance, Durán-Rosal et al. [2] proposed using the evolutionary unit neural network (EPUNN) and the linear model as the input portion to reconstruct the data to meet the constantly changing data flow.

The literature includes three main approaches to building predictive models for ocean marine data: statistical, deep learning, and machine learning approaches. Popular ocean wave models such as wave model (WAM), WAVEWATCH III model, and Simulating Wave Nearshore (SWAN) were forecasted conventionally. However, researchers commenced using ML to predict ocean waves [3–8].

Using the SWAN wave model, an efficient multilayer perceptron algorithm was proposed to forecast lake waves in Michigan [3]. This algorithm estimated relevant wave features such as peak periods and heights in different weather conditions. The Artificial Neural Network (ANN) and Support Vector Machine (SVM) models are both employed for wave prediction purposes. The SVM model, on the other hand, was shown to be more accurate than ANN, with slightly lower error than the ANN model. Besides, it is characterized by its fewer parameters and faster computation time. In [7], Wu et al. proposed and developed a physics-based machine learning (PBML) model that combines the physics-based wave model with a machine learning technique for multistep-ahead wave forecasting for marine operations. Bento et al. developed a new methodology based on deep neural network to predict the generated electrical power of ocean wave energy systems [8]. Despite all of these efforts, the literature has no comparison of the aforementioned three approaches. This comparison should reveal the performance gap between these approaches.

This paper aims to predict the SST and the SWH for the Korea Hydrographic and Oceanographic Agency dataset. The proposed work is motivated by comparing the statistical, machine learning, and deep learning models to understand the performance gap of these models. The results of this work should provide scientific evidence on which model fits better the marine data. To anticipate the marine features efficiently, the employed deep learning model combines the gated recurrent units (GRU) with the regular neural network. In the proposed architecture, the GRU layer is preceded by an input layer and followed by a fully connected layer. As a result, the predicted values can be produced from the output layer. To our knowledge, this is the first use of a GRU model architecture for forecasting SST and SWH. Besides, four different ML models have been utilized in the current study, namely, Linear Regression (LR), Decision Tree (DT), Support Vector Machine (SVM), and Random Forest (RF) Regressor. In addition, a statistical model has been applied to the same dataset to forecast both the temperature and the wave height, which is the Autoregressive Integrated Moving Average (ARIMA) model. Consequently, the prediction problem is treated as a regression problem in machine learning techniques. However, ARIMA and GRU frame with this prediction problem as a time series problem. The main contributions of this paper can be summarized as follows.

1. To the best of the authors' knowledge, we proposed the first GRU model architecture for predicting SST and SWH.
2. A comparison among statistical, machine learning, and deep learning models is held to evaluate which model fits this prediction issue the best and to understand the performance gap between these models
3. To our knowledge, this is considered the first time to predict the SST and SWH features for the Korea Hydrographic and Oceanographic Agency.

The proposed comparison is generic. Thus, it can be applied to other marine data such as wind direction, salinity, and water current predictions. In addition, the comparison of the statistical, machine learning, and deep learning models can be extended to other similar applications such as climate forecasting, navigation and ship traffic, or fishing activities.

The rest of this paper is organized as follows. Background regarding the GRU architecture and the machine learning algorithms are briefly explained in Section 2.  Section 3 discusses the related work. The proposed methodology and the system block diagram are presented in Section 4. The experimental results and discussions are detailed in Section 5. Finally, Section 6 summarizes the conclusion.

## 2. Background

### 2.1. Deep Learning-Based Prediction: GRU

Deep learning algorithms, particularly recurrent neural networks (RNNs), have been proven successful in a variety of applications, including time series forecasting [9, 10]. The RNN is a powerful model that can learn a wide range of complex relationships from an arbitrarily long sequence of data and has been used to effectively solve many problems [11–13]. However, two well-known problems were raised because of the depth of RNN, namely, exploding and vanishing gradient. To address the difficulties mentioned above, two variations of the recurrent model were introduced (i.e., GRU [14] and LSTM [15]). The GRU and LSTM architectures are similar in design, and both contain gating techniques for controlling the flow of data through the unit. Despite this, due to its complicated structure, the LSTM takes a long time to train and converge. GRU-DNN is simpler than LSTM and has a less sophisticated architecture. As a result, it is faster to train than LSTM [16].

**Figure 1:**
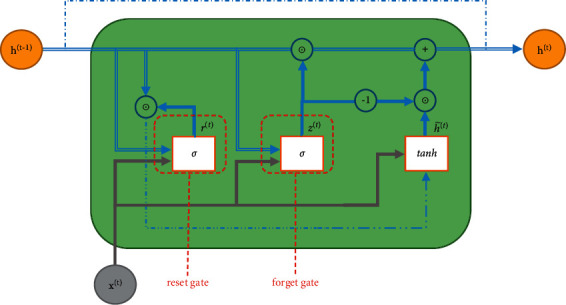
GRU-DNN cell architecture.

In the GRU model, recurrent units capture patterns and dependencies across time spans. Unlike the LSTM cell, GRU does not have a unique memory gate, making it more efficient and quicker in data training. A standard GRU architecture cell is depicted in Figure 1.

A GRU model is made up of a set of cells. There are two gates and a state vector in each cell. In any cell of a GRU model, there are two gate types: update *z*^(*t*)^ and reset *r*^(*t*)^gates, with *h*^(*t*)^denoting the hidden state vector for the current time point *t*. Each gate is made up of a single-layer neural network. The following equations illustrate the architecture of GRU cell equations (1)–(4). The hidden state of the previous cell (denoted as *h*^(*t* − 1)^) and the current input sequence vector (denoted as *x*^(*t*)^) are given to the cell as an input. The hidden state (denoted as *h*^(*t*)^) is the cell output.

(1) $$\begin{array}{c} z^{\left(t\right)}=\sigma \left(W_{z}x^{\left(t\right)}+U_{z}h^{\left(t-1\right)}+b_{z}\right), \end{array}$$

(2) $$\begin{array}{c} r^{\left(t\right)}=\sigma \left(W_{r}x^{\left(t\right)}+U_{r}h^{\left(t-1\right)}+b_{r}\right), \end{array}$$

(3) $$\begin{array}{c} {\tilde{h}}^{\left(t\right)}=\tanh\left(W_{h}x^{\left(t\right)}+U_{h}\left(h^{\left(t-1\right)}⊙r^{\left(t\right)}\right)+b_{h}\right), \end{array}$$

(4) $$\begin{array}{c} h^{\left(t\right)}=z^{\left(t\right)}⊙h^{\left(t-1\right)}+\left(1-z^{\left(t\right)}\right)⊙{\tilde{h}}^{\left(t\right)}, \end{array}$$

where ⊙ denotes elementwise multiplication, *σ*(.) and tanh(.) denote the sigmoid and hyperbolic tangent activation functions of the neural network (NN), respectively, ${\tilde{h}}^{\left(t\right)}$ denotes the candidate hidden state, *W*_*z*_, *W*_*r*_, and *W*_*h*_ denote the cell model's weight matrices for the feedforward neural networks, *U*_*z*_, *U*_*r*_, and *U*_*h*_ denote the cell model's weight matrices for recurrent neural networks, and the model biases are *b*_*z*_, *b*_*r*_, and *b*_*h*_.

The output of a GRU cell (*h*^(*t*)^, provided by equation (4)) is a linear interpolation between the current candidate state ${\tilde{h}}^{\left(t\right)}$ and the prior concealed state *h*^(*t* − 1)^. This type of linear interpolation is mostly used to learn long-term dependencies. More precisely, as *z*^(*t*)^tends to 1, the previous hidden state remains unchanged and may be maintained for a few time steps. On the other hand, as *z*^(*t*)^ goes to 0, the cell output equals the value of the candidate state ${\tilde{h}}^{\left(t\right)}$, which is extremely reliant on the current input and prior hidden state. The candidate state ${\tilde{h}}^{\left(t\right)}$ is also reliant on the reset gate *r*^(*t*)^, which compels the cell to omit or preserve the last hidden states.

### 2.2. Machine Learning Prediction Models

Machine learning includes three main categories, namely, supervised learning (e.g., classification or regression), unsupervised learning (e.g., clustering or association), and reinforcement learning (e.g., reward-based). In this work, we focus on the first category of the ML field, i.e., supervised learning [17]. The ultimate goal of the machine learning field is to design models/programs which enable computer systems to mimic the learning process of human beings from the available data. Any ML-based system consists of three components, namely, data, models, and learning. The main task of designing an ML system is to fit the data to the model by tuning the model's hyperparameters. This task is called model training; it is accomplished using hypotheses based on performance criteria. Hyperparameter optimization aims to determine the ideal collection of the corresponding hyperparameters for a ML model. Identifying the optimal configuration of hyperparameter values for a predictive model has a direct effect on the models' performance and the tested dataset. While hyperparameter tuning is a crucial step in the model training process to ensure a successful ML application, it is a compute-intensive procedure. This is because of the large number of possible combinations to test and the computational resources required [18, 19].

The regression task in the ML field is considered one of the fundamental tasks. Designing an ML-based regressor includes utilizing mathematical techniques to predict the continuous output variable *Y* based on the value of one or more input variables *X*. Linear Regression is the simplest regression analysis for the sake of predicting the output based on historical data. Hence, the life cycle of any ML model contains four main stages which are selecting the training data, choosing the target function, the representation for the target function, and selecting a function approximation methodology.

### 2.3. Statistical Predictive Models

Time series modelling is a dynamic research topic that tries to gather and examine historical data of a time series in order to construct a model that accurately describes the series' inherent structure [20]. This model is then used to forecast future values for the series, taking into consideration that proper model fitting is required for good time series forecasting.

Researchers have been focused on linear models for the past few decades because they are simple to understand and apply. In linear models, the future values are constrained to be linear functions of past data. The ARIMA [21–25] model is one of the most popular and widely used linear stochastic time series models. Other models, such as the Autoregressive (AR) [21, 24–26], Moving Average (MA) [21, 24, 25], and Autoregressive Moving Average (ARMA) [21, 23–25], are subclasses of the ARIMA model.

Many time series, including those connected to socioeconomic [24] and business, exhibit nonstationary behaviour in practice. Time series with trends and seasonal patterns are also nonstationary [27, 28]. The ARMA model can only be used for stationary time series data; they are inadequate to describe nonstationary time series accurately. As a result, the ARIMA model emerged to take into account nonstationarity.

In the process of designing an ARIMA model, a nonstationary time series can be rendered stationary by applying finite-difference of the data points. If a time series is integrated of order 1, expressed as *I*(1), it will be stationary after the first differentiation and expressed as *I*(0). In general, if a time series is *I*(*d*), it becomes a stationary series *I*(0) after differentiation at *d* times [29].

An ARIMA model is denoted as ARIMA(*p*, *d*, *q*), where *p* is the number of autoregressive terms, *d* is the number of differences, and *q* is the number of moving averages [30].

## 3. Related Work

### 3.1. SST Forecasting

SST is a critical parameter to be forecasted in the marine environment since it can affect a variety of events such as sports, fishing, marine ecology, and weather forecasting. Hence, predicting the SST in both the short and long term is an active topic that has recently drawn researchers' attention. A prediction technique based on Support Vector Machine (SVM) has been introduced for determining SST in the Tropical Atlantic region [31]. The utilized dataset in [31] is considered the raw data feed to the SVM model and is collected from two PIRATA buoys (placed at 8°N 38°W and 10°S 10°W), employed in this study. The authors' proposed system extends the work proposed in [32], which uses the same PIRATA dataset.

Normally, the sea surface temperature can be predicted both in the short term (i.e., a few days) and in the long term (i.e., weekly and monthly). This problem can be expressed as a problem of time series regression. Hence, the same as [33], the long short-term memory (LSTM) can be used to forecast the SST. In this study [33], the time series is initially modelled using an LSTM layer. Afterward, a fully connected layer is employed to handle the output of the LSTM layer to predict SST. In [33], the authors proposed making use of the sea surface temperature values for the Baohai Chinese coastal seas.

A method for forecasting daily sea surface temperatures over the short and medium term has been developed using a case study in the East China Sea using 36 years of satellite time series data in [34]. Rather than the actual sea surface temperature, this approach used the historical time series satellite anomaly. A combined long short-term memory (LSTM) and AdaBoost ensemble learning model is employed in this machine learning system to achieve higher accuracy and hence adequate temperature prediction. Another integrated Deep Gated Recurrent Unit and conventional neural network (DGCnetwork) was also applied on the East China Sea and the Yellow Sea dataset [35]. The deep GRU and the convolutional layers are used to extract the deep hidden temporal features and spatial properties of SST data, respectively. This technique was successful in achieving a 98 percent accuracy rate.

A hybrid approach has been introduced in [36] that integrates both numerical and data-driven methodologies. This mitigates the drawbacks of just applying the numerical forecast to the sea surface, which exhibits huge variances when applied to a site-specific case study and decreased accuracy for long-term prediction. This study used deep learning neural networks along with numerical estimators at different locations in India for daily, weekly, and monthly forecasting. To begin, conventional neural networks are implemented for prediction, followed by the application of the LSTM across all timescales. The LSTM is sensitive to gap lengths and has higher data extraction capability compared to the linear methods. A comparison to the linear system (ARIMAX) [21] established that linear models could not perfectly deal with broad and varying time horizons.

In [37], the authors proposed building a predictive model for predicting the SST of the entire China Sea. They utilized collected data over 12 months. They proposed a deep learning model using the LSTM architecture for the task of SST prediction. Their work proposed splitting the gathered data into two parts, namely, SST anomalies and SST means. Then, they used each data split for training the proposed LSTM model. Besides, they proposed using a self-organizing feature map (SOM) neural network to classify different subregions; these classifier model results are used to enhance the SST forecasting accuracy.

### 3.2. SWH Forecasting

In [38], the authors collected the marine data from three different regions, namely, (1) Gulf of Mexico, (2) Korean region, and (3) UK region. The utilized datasets are gathered four times per day (i.e., every six hours) from 13 stations scattered over these three areas. The proposed model predicts the daily SWH at 12 a.m. in each station. The authors proposed two models and compared them against the extreme learning machines (ELM) and Support Vector Regression (SVR) models. The obtained results outline a significant performance gap between the proposed models and ELM and SVR. The proposed models outperformed the standard ELM and SVR.

Wave height forecasting is crucial for various coastal engineering applications. In [6], Mahjoobi et al. employed support vector machines (SVR) and multilayer perceptron (MLP) for forecasting significant wave height. For that purpose, the authors utilized data set of Lake Michigan where wind speed is used to predict wave height values. Similarly, Shamshirband et al. [39] used wind data to forecast wave height using data gathered from two different locations of the Persian Gulf. The experiments are performed using a numerical model (i.e., Simulating Waves Nearshore (SWAN)) and ML-based models (i.e., artificial neural networks (ANN), extreme learning machines (ELM), and SVR) for wave height modelling.

Deep learning technology is increasingly being utilized to forecast time series data in a variety of sectors. Authors of [40] used a conditional restricted Boltzmann machine (CRBM), including temporary information in the classical deep belief network (DBN), to predict the significant wave height. This prediction used key model parameters derived by applying the particle swarm optimization (PSO) algorithm to the wave data. For the entire prediction error, CE and RMSE are employed as evaluation metrics. This research evaluated the model's efficiency using two different statistical measures, namely, RMSE and the Nash–Sutcliffe coefficient of efficiency (CE).

Forecasting significant wave height (SWH) is an essential technique in offshore and coastal engineering. Due to the randomness and fluctuation characteristics of waves, precise prediction of the SWH is a difficult task. The authors of [41] use a new deep learning algorithm called the gated recurrent unit network (GRU) to forecast SWH through different time durations. The wind speed data for the SWH were gathered from six buoy stations through various sites in the Taiwan Strait and its nearby waters and were used as input for the algorithm. Three different statistical metrics, including RMSE, coefficient of correlation (R), and an index of agreement (IA), have been used in this paper to evaluate the algorithm's efficiency. The paper presented that the GRU can produce more accurate forecasting values and capture the overall data trend.

The discussion of the existing methods shows that there is no comparison of the different methods (i.e., machine learning, deep learning, and statistical models) in terms of prediction accuracy. Thus, there is a need to study the performance gap of these methods for predicting marine data.

## 4. Methodology

**Figure 2:**
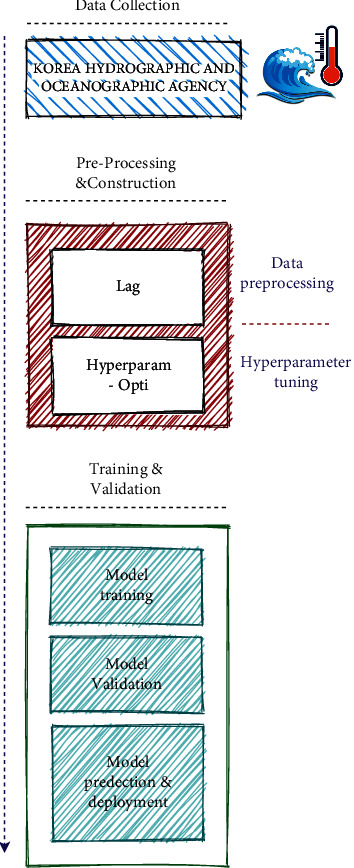
The block diagram of the proposed system.

In this work, we proposed a set of predictive models which are based on three different approaches (i.e., machine learning, deep learning, and statistical). The proposed generic framework is composed of three stages: data gathering, preprocessing, and machine learning model deployment. This system makes use of the Korea Hydrographic and Oceanographic Agency dataset (available at: https://www.khoa.go.kr/eng/). The data collection stage targets collecting vital marine features via installed sea sensors. The obtained data is preprocessed and fine-tuned for the purpose of filtering. Finally, the dataset is subjected to training and tuning the parameters of the predictive models. The overall framework block diagram for estimating both SWH and SST is presented in Figure 2.

Regarding the raw data, first, we proposed preprocessing this huge dataset to address the noisy and missing values that are commonly encountered during data acquisition. Second, prior data is used to predict the next step, known as the lag method [42]. Thus, the lag is applied to the significant features, which are temperature and wave height, to forecast the next values. Basically, the lag value is not determined until we explore various lag values and then observe the resulting accuracy rates. Finally, the best lag value with the highest accuracy is selected. This section exposes the details of the proposed GRU-DNN model, the process of tuning the machine learning models, and the statistical ARIMA model.

### 4.1. Stacked GRU-DNN Model

The first model is a proposed GRU-DNN model architecture (a deep learning model). Different model architectures result in different prediction rates. Thus, the main challenge was to find the best GRU architecture that fits the data at hand. The proposed Stacked GRU-DNN is a flexible custom model, where its architecture is varied according to the training data. In other words, the proposed model has no specific architecture, and its hyperparameters are obtained during the hyperparameter optimization process.

**Figure 3:**
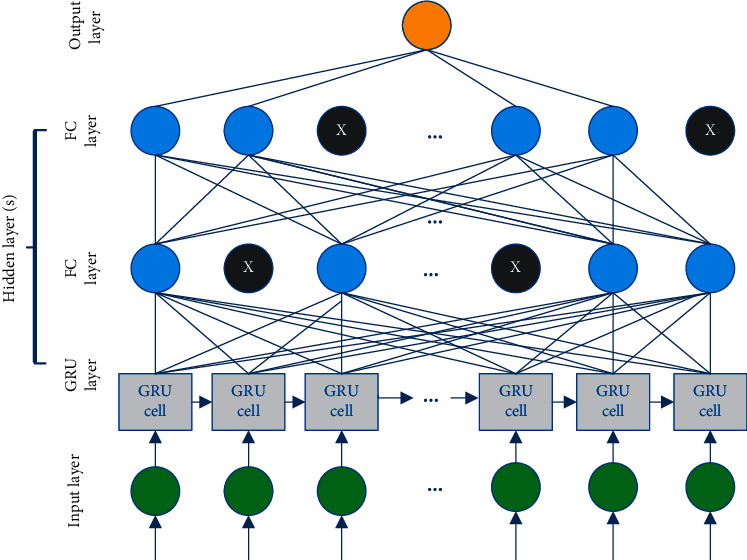
Stacked GRU-DNN model.

The proposed GRU-DNN stacking model is represented as seen in Figure 3. As depicted in Figure 3, the proposed model consists of an input layer that receives model input, a GRU layer, a fully connected layer(s), and, lastly, a single neuron output layer that produces the forecasted result. We aim at using the proposed model structure to use a recurrent layer that can learn and model time series patterns in data, besides the additional fully connected layer(s) that recombine the extracted representation learned through previous layers and get extra representations of more levels of abstraction.

Practically, over/underfitting difficulties in neural network models are caused by the neural network model's excessive/insufficient training epochs [43]. As a result, one possible solution to the DL-based model's over/underfitting concerns is to apply the early stopping strategy [44], which is used to cease training when generalisation performance starts to degrade for a number of epochs. To track the generalisation performance, in the proposed model, the training data is separated into training and validation groups.

The dropout approach [45] is another way to deal with the overfitting problem. Dropout is a regularisation strategy that allows you to train neural networks with alternative topologies in parallel by randomly dropping out a certain proportion of layer neurons. Dropout is indicated by the black neurons in the fully connected layers, as seen in Figure 3.

One of the well-known adaptive optimization algorithms which have been shown to be effective in solving practical DL issues is the Adam optimizer [46]. DL model uses the Mean Square Error (MSE) loss function, which is provided by equation (5). That is, the proposed GRU-DNN model is trained with the goal of minimizing the loss function given a training data {(*X*_*i*_, *Y*_*i*_)}_*i*=1_^*N*^ of *N* observations.

(5) $$\begin{array}{c} \underset{w}{\min}\frac{1}{N}\sum_{j=1}^{N}\left\{Y_{j}-F{\left(X_{j},w\right)}_{2}\right\}, \end{array}$$

where *w* signifies the network coefficient, *F* : ℝ^*k*^⟶ℝ^1^ is the neural network flow, and *k* denotes the size of the input vector (i.e., number of lag features).

#### 4.1.1. GRU-DNN Hyperparameter Optimization

The optimization of the proposed model hyperparameters is a part of machine learning methods. The model parameters (coefficients) utilized to govern the training task are as hyperparameters. Such parameters (e.g., learning rate, number of layers/neurons of a network/layer, lag order of ARIMA model, etc.) must be fine-tuned in order to obtain good fitting/generalisation of the model in a process known as hyperparameter tuning.

In the proposed model, the optimal model hyperparameters are obtained utilizing a distributed asynchronous hyperparameter optimization approach [47]. Therefore, for parameter finding and optimization, we used the Tree Parzen Estimator (TPE) [47] method in the Hyperopt package (available at http://hyperopt.github.io/hyperopt/).  Table 1 shows the proposed GRU-DNN model hyperparameters and their search spaces for determining the best model hyperparameter values.

### 4.2. Machine Learning Models

One of the main obstacles for designing machine learning models is tuning the hyperparameters of the model. This is because different hyperparameter values can lead to different accuracy levels. In the proposed system, four machine learning models are employed, which are Linear Regression, Support Vector Regression (SVR), Decision Tree (DT), and Random Forest (RF). Each learning model is subjected to a grid search in order to achieve the optimal parameter tuning. The hyperparameters are essential for being one of the primary sources that influence the behaviour of a machine learning model in general. Hence, determining the optimal hyperparameters combination is a critical goal which reduces a predefined loss function and produces better outcomes. For instance, the degree, kernel, epsilon, and gamma are all adjusted in SVR to reach the highest accuracy. In RF, however, grid search is applied to determine the optimal hyperparameters (i.e., *max_features, min_samples_leaf, number of estimators, and min_samples_split*). Similarly, the max depth and criterion are the hyperparameters of the DT.  Table 2 shows the optimized values for the hyperparameters of the employed four ML models. In addition, for ML models, we tuned the number of the lag features which is considered as a hyperparameter that requires optimization. Hence, the optimized lag features transform the time series problems into supervised ML ones.

### 4.3. ARIMA Model

We proposed a new ARIMA model to solve the proposed problem. In the proposed model, we used the autocorrelation function (ACF) and partial autocorrelation function (PACF) to get the ARIMA parameters such as *p*, *d*, and *q*. By viewing the ACF scheme at *d* = 1, the parameter *q* may be determined once these procedures have been completed. The first lag is essential, while the second lag is not significant. Thus, the MA term has a value of 1 and is represented by *q*. The ACF for the second differencing means the lag advances quickly towards the far negative zone, implying that the series may have been overdifferences. As a result, even though the series is not entirely stationary, we set the order of differencing to 1. In the end, the last parameter of the ARIMA model is *p*, which may be determined by examining the PACF diagram.

We demonstrated how to decompose a time series for checking the presence of a seasonal component by decomposing one sample (i.e., minimum of wave height-series of ocean marine necessary parameters). We used the *seasonaldecompose* function from the *statsmodels* library in the Python program to decompose the time series. The decomposition shows that the minimum time series of ocean waves has a seasonal part for one marine feature type. Since the time series has a definite seasonality, the SARIMA, which uses seasonal differencing including characteristics (P, D, Q, and S), is the way to proceed. We proposed utilizing a grid search for the P, D, and Q parameters to find the best seasonal parameters within various values (e.g., 0, 1, 2, and 3). In contrast, the search space for parameter S encompassed 6, 12, and 24. These parameters are determined via a grid search, which entails testing numerous seasonal parameters and reporting the combination of parameter values with the highest accuracy metrics scores.

## 5. Experimental Results

### 5.1. Experimental Setup

The experiments were performed on a computer running 64-bit Linux OS with two 2.3 GHz Intel 8-core processors. All of the utilized predictive models were implemented in the Python programming language version 3.8.5. Moreover, deep learning model (i.e., GRU-DNN) implementation is performed using Keras (Available at https://keras.io) and Tensorflow [48] version 2.3.1. Furthermore, the following libraries are utilized in our work: Hyperopt [47], statsmodels [49], Scikit-Learn [50], pandas [51], Numpy [52], and Matplotlib [53].

### 5.2. Dataset

As previously stated, the Korea Hydrographic and Oceanographic Agency dataset includes gathered real-time observed marine data [54]. This data is updated every 30 minutes from the located underwater network at latitude 34.223611 and longitude 128.4205552. Relevant data, such as salinity, temperature, wave height, water current, and surface direction, is sensed and forwarded to central sea buoys through surrounding sensors placed in each sector. Afterwards, the buoys filter pertinent data before transmitting it to the above-ground base station. We utilized the data from ten different stations/buoys. In this work, we collected the data from ten different buoys at different locations.

### 5.3. Evaluation Metrics

Evaluating the machine learning algorithms that are being used is a critical component of any proposed system. Four assessment metrics are used to determine the correctness of this system. The mean absolute error (MAE) is one of these metrics that estimate the average of the difference between original and forecasted values. Hence, we can determine how far the predictions differ from the actual data. Mathematically, MAE is represented as follows:

(6) $$\begin{array}{c} \text{MAE}=\frac{1}{N}\overset{N}{\underset{i=1}{\sum }}\left|Y_{i}-{\hat{Y}}_{i}\right|, \end{array}$$

where *Y*_*i*_ denotes the true values, ${\hat{Y}}_{i}$ represents the predicted values, and *N* represents the number of observations.

The mean squared error (MSE) and the root mean square error (RMSE), on the other hand, are also deployed to assess the accuracy of regression problems. The MSE is the average of the square of the difference between actual and predicted values. The MSE is expressed as

(7) $$\begin{array}{c} \text{MSE}=\frac{1}{N}\overset{N}{\underset{i=1}{\sum }}{\left(Y_{i}-{\hat{Y}}_{i}\right)}^{2}. \end{array}$$

Basically, when we consider the square of the error, the effect of larger errors becomes more evident. The standard deviation of these errors, which occur during the prediction process, is known as the RMSE. In this case, we take the root of the values into account while calculating the accuracy. The RMSE is calculated as

(8) $$\begin{array}{c} \text{RMSE}=\sqrt{\frac{1}{N}\overset{N}{\underset{i=1}{\sum }}{\left(Y_{i}-{\hat{Y}}_{i}\right)}^{2}}. \end{array}$$

Finally, the R_Squared (*R*^2^) value, which ranges from 0 to 1, reflects whether a model fits a given dataset. Similarly, it quantifies the closeness to the regression line with respect to the actual values. The following formula can be used to calculate the *R*^2^ metric:

(9) $$\begin{array}{c} R^{2}=1-\frac{\sum_{i=1}^{N}{\left(Y_{i}-{\hat{Y}}_{i}\right)}^{2}}{\sum_{i=1}^{N}{\left(Y_{i}-\bar{Y}\right)}^{2}}, \end{array}$$

where $\bar{Y}$ denotes the mean true values.

### 5.4. Results

The first point of comparison is the performance of the implemented models over the utilized four evaluation metrics discussed in Subsection 5.3. The obtained results are listed in Table 3 and 4 for predicting SST and SWH, respectively. The reported values are the mean and standard deviation predicting of the utilized ten stations. In Table 3, the GRU-DNN and SVR models outperform the other models over the four evaluation metrics, where the GRU-DNN model was slightly better than the SVR model. The worst model was the ARIMA model. This is because the proposed model predicts 1000 steps ahead. The ARIMA model performance degrades as the value of step ahead prediction increases.

For the SWH forecasting, the listed results in Table 4 show that the RF and GRU-DNN outperformed the other predictive models. The RF model slightly outperforms the GRU-DNN model. Again, the ARIMA model failed in achieving competitive results. This is linked to the same reason for the large size of the step ahead prediction.

**Figure 4:**
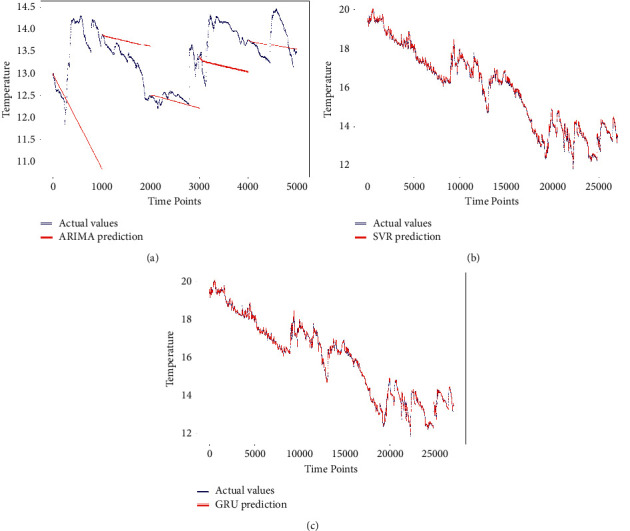
The actual SST vs. the forecasted SST, (a) ARIMA, (b) SVR, (c) GRU-DNN.

**Figure 5:**
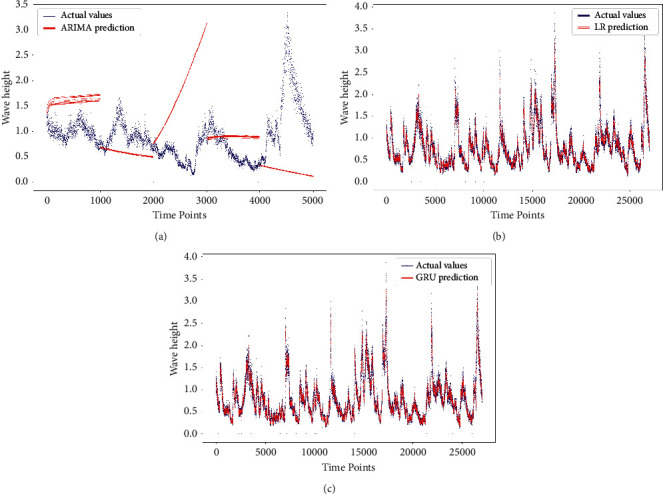
The actual SWH vs. the forecasted SWH, (a) ARIMA, (b) LR, (c) GRU-DNN.

The second point of comparison is the visual illustration. In Figures 4 and 5, we have the actual values against the predicted values for the SST and SWH, respectively. Thus, deep learning, machine learning, and statistical models are contributing with a subfigure in Figures 4 and 5, where the machine learning models are represented by the best performing model. The scatter plots in Figures 4 and 5 show a perfect fit for the deep learning and machine learning models in 4(b), 4(c), 5(b), and 5(c).

### 5.5. GRU-DNN Hyperparameter Analysis

**Figure 6:**
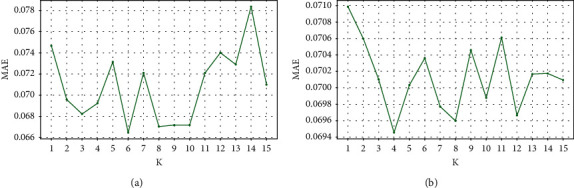
Model performance of using a different number of lag features. (a) Water temperature. (b) Significant wave height.

**Figure 7:**
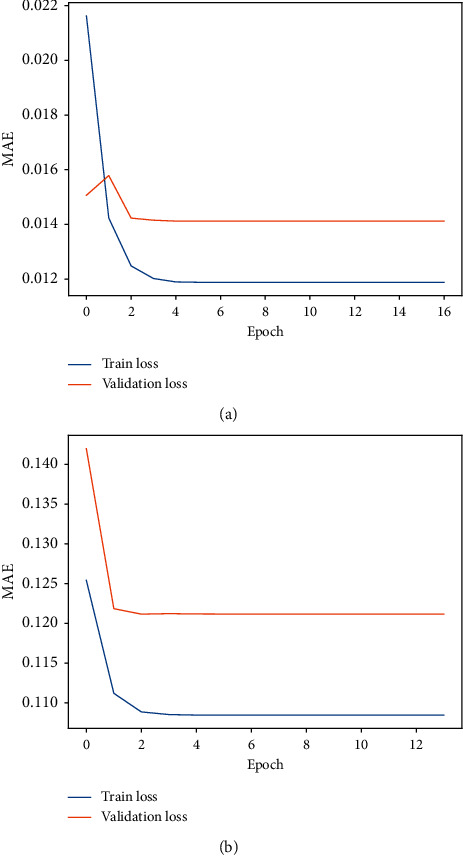
Train and validation loss. (a) Water temperature. (b) Significant wave height.

GRU-DNN model is trained in a supervised learning fashion using lag features (i.e., using *K* previous observations), where *K* denotes the number of previous observations used in the training and forecasting task. Typically, *K* is considered as a hyperparameter that needs to be optimized. Therefore, we performed a grid search method to obtain the optimal *K* value.  Figure 6 depicts the grid search for different values of *K* hyperparameter over search space ranges from 1 to 15. Specifically, Figure 6(a) presents the model performance for water temperature forecasting using various *K* values, where *K*=6 achieves the lowest MAE error. Similarly, *K*=4 is the optimal value for significant wave height forecasting as shown in Figure 6(b). It is noteworthy that the experiments presented in Figure 7 are for the first dataset of each forecasting problem, assuming that the rest of the datasets have similar behaviour.

## 6. Conclusions

The huge advances of ocean observation systems yield a large amount of marine data. Thus, this huge data can be utilized to train predictive models to forecast future marine data. In this work, we proposed predicting SST and SWH by implementing machine learning, deep learning, and statistical techniques models. In turn, a comparison of these three approaches is conducted on different evaluation metrics, namely, MAE, MSE, RMSE, and *R*^2^. The comparison utilized a real dataset obtained from the Korea Hydrographic and Oceanographic Agency. The simulation results show that the machine learning models are slightly better than the implemented deep learning model. The best model that predicted the SST was the DTs, while the Linear Regression model was the best model for SWH forecasting. The statistical model (i.e., ARIMA) has been confirmed to have the worst performance.

## Figures and Tables

**Table 1:** The search space of the GRU-DNN model hyperparameters.

Hyperparameters	Value
No. of GRU cells	[4, 8, 16]
No. of FC layers	[1, 2]
No. of FC layers' units	[4, 8, 16]
Hidden layers activation	[Relu, Linear]
Batch size	[4, 8, 16]
Droupout rate of FC layers	[0.0, 0.1, 0.2]

**Table 2:** The hyperparameters values of the ML models.

Decision tree		
Best parameter	Significant wave height	Water temperature
Criterion	Mae	Mae
max_depth	7	10
max_features	Auto	Log2
min_samples_split	4	Default
Random forest		
Bootstrap	True	True
max_depth	100	90
max_features	3	2
min_samples_leaf	5	5
min_samples_split	8	12
n_estimators	200	200

SVR		
C	100	1
Degree	1	1
Gamma	Auto	Default
Epsilon	Default	0.2
Kernel	Poly	Poly

**Table 3:** The evaluation metrics for the SST prediction of different models.

Model	MAE	MSE	RMSE	*R* ^2^ (%)
ARIMA	0.9601 ± 0.4270	1.6392 ± 1.0761	1.1958 ± 0.4820	NA
D-tree	0.0772 ± 0.0170	0.0272 ± 0.0121	0.1346 ± 0.0318	99.49 ± 0.21
RF	0.0607 ± 0.0144	0.0190 ± 0.0106	0.1076 ± 0.0287	99.63 ± 0.19
SVR	0.0515 ± 0.0096	0.0138 ± 0.0072	0.0954 ± 0.0230	99.73 ± 0.13
LR	0.0651 ± 0.0144	0.0226 ± 0.0088	0.1302 ± 0.0251	99.88 ± 0.03
GRU-DNN	0.0498 ± 0.0353	0.0136 ± 0.0219	0.0934 ± 0.0738	99.74 ± 0.40

**Table 4:** The evaluation metrics for the SWH prediction of different models.

Model	MAE	MSE	RMSE	*R* ^2^ (%)
ARIMA	0.4786 ± 0.2648	0.5060 ± 42.26	0.6311 ± 0.3460	NA
D-tree	0.0709 ± 0.0099	0.0203 ± 0.0061	0.1282 ± 0.0207	94.13 ± 1.23
RF	0.0646 ± 0.0096	0.0174 ± 0.0061	0.1164 ± 0.0207	95.01 ± 1.25
SVR	0.0698 ± 0.0087	0.0198 ± 0.0066	0.1259 ± 0.0210	92.96 ± 2.55
LR	0.0681 ± 0.0094	0.0196 ± 0.0065	0.1248 ± 0.0213	94.42 ± 1.20
GRU-DNN	0.0673 ± 0.0293	0.0186 ± 0.0195	0.1218 ± 0.0652	94.63 ± 3.77

## Data Availability

The utilized dataset in this work is available online https://www.khoa.go.kr/eng/.
